# Dietary Flavonoids for Immunoregulation and Cancer: Food Design for Targeting Disease

**DOI:** 10.3390/antiox8070202

**Published:** 2019-06-29

**Authors:** Jennifer H. Ahn-Jarvis, Arti Parihar, Andrea I. Doseff

**Affiliations:** 1 Food Innovations and Health, Quadram Institute Bioscience, Norwich NR4 7UQ, UK; 2 Department of Science, Bellingham Technical College, Bellingham, WA 98225, USA; 3 Department of Physiology, Michigan State University, East Lansing, MI 48864, USA; 4 Department of Pharmacology & Toxicology, East Lansing, MI 48864, USA

**Keywords:** flavonoids, functional foods, antioxidants, chemoprevention, apoptosis, inflammation, immune-regulation, tumor associated macrophages, medical foods, clinical trials

## Abstract

Flavonoids, one of the most abundant phytochemicals in a diet rich in fruits and vegetables, have been recognized as possessing anti-proliferative, antioxidant, anti-inflammatory, and estrogenic activities. Numerous cellular and animal-based studies show that flavonoids can function as antioxidants by preventing DNA damage and scavenging reactive oxygen radicals, inhibiting formation of DNA adducts, enhancing DNA repair, interfering with chemical damage by induction of Phase II enzymes, and modifying signaling pathways. Recent evidence also shows their ability to regulate the immune system. However, findings from clinical trials have been mixed with no clear consensus on dose, frequency, or type of flavonoids best suited to elicit many of the beneficial effects. Delivery of these bioactive compounds to their biological targets through “targeted designed” food processing strategies is critical to reach effective concentration in vivo. Thus, the identification of novel approaches that optimize flavonoid bioavailability is essential for their successful clinical application. In this review, we discuss the relevance of increasing flavonoid bioavailability, by agricultural engineering and “targeted food design” in the context of the immune system and cancer.

## 1. Introduction

Advances in plant genetics, horticultural technology, and food processing have facilitated the development of functional foods or medical foods for disease prevention and as adjuvant therapy by enhancing the content of bioactive compounds in crops and improving their bioavailability in food products. The potential advantages of a food-based approach in disease management include: (1) whole food ingredients containing complex mixtures of bioactive phytochemicals that impact multiple targets to enhance immune function [1]; (2) complex mixture of bioactive phytochemicals may provide additive and/or synergistic activities [2,3]; (3) easy administration and usually non-toxic side effects provide unique opportunities to improve quality of life of patients; (4) cost-effective alternatives that can be used alone or in combination with currently available standard of care in the prevention and treatment of cancer.

Numerous epidemiological, cellular, and animal studies have substantiated the potential health benefits of flavonoids. Recent meta-analyses have reported the inverse relationship between dietary intake of flavonoids with cardiovascular disease (CVD) mortality [4], CVD [5], diabetes [6], mental illness [7] and risk of several cancers [8]. Yet, the evidence from dietary intervention trials is at best modest [9,10]. Unlike cellular and animal studies, the doses administered, duration of exposure, and precise staging of cancer targets remain unclear in the limited clinical studies so far available. Epidemiological evidence relies on case-control studies and estimates on flavonoid intake within a specific geographical region where multifactorial differences such as lifestyle, environment, and demographics are inimitable in clinical trials. Clinical application of flavonoids-rich foods presents unique opportunities to decipher the role of dietary flavonoids in disease control. To date, there is no consensus on standardized doses or sources of flavonoids for clinical studies. Moreover, how the bioavailability and biotransformation by the gut microbiota affect their nutraceutical activities in humans remains poorly studied. Targeted food design and innovative food processing strategies can be used to create functional foods to control primary tumors and metastasis, either by directly targeting cancer cells or other cells found in the tumor microenvironment (TME). Intriguing clinical studies in the past few years show new advances in the field of cancer immunotherapy. Flavonoids have been shown to effectively modulate the immune response. Hence, their potential to regulate the recruitment of immune cells into the TME and/or overcome tumor immunosuppressive conditions offer critical opportunities to use flavonoids alone or in combination with the current standard of care for the prevention and treatment of cancer. Therefore, this review focuses on dietary flavonoids highlighting specifically glycoside forms which are frequently found in foods. Flavanols, abundant in tea, cocoa, and fruit skins, have been omitted since they undergo very different processing and metabolism, but comprehensive reviews can be found else were [11,12,13,14,15]. We present strategies of food design to enhance flavonoid bioactivity and use as functional foods for the prevention and treatment of cancer with special emphasis, when possible, in immunoregulation.

## 2. Flavonoids Chemical Structure and Function: From Plants to Cancer Therapy

**Figure 1:**
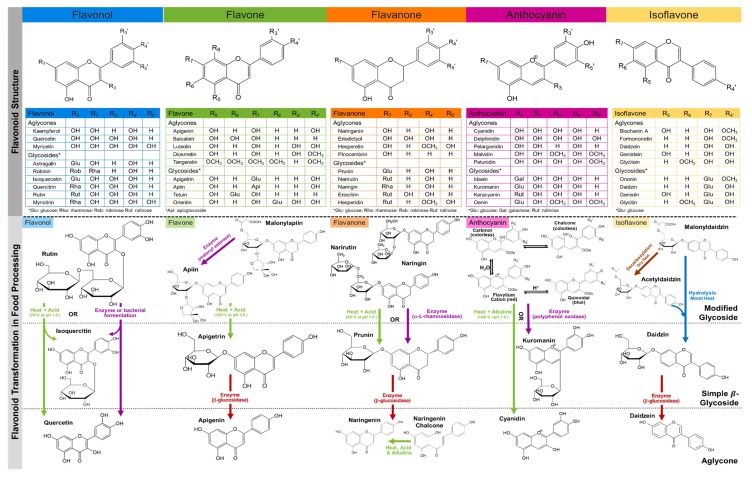
Flavonoid structures organized by their families and an example of their structural transformations during food processing. Flavonols have been omitted as they are not found in food as glycosides.

Flavonoids are low-molecular weight heterocyclic compounds synthesized from the amino acid products of the shikimate pathway. Structurally, flavonoids are comprised of two benzene rings (A and B) bridged together by a heterocyclic pyrone (C) and they are classified based on their structure in different groups including: flavonols, flavones, flavanones, anthocyanins, and isoflavones, as shown in Figure 1. Flavonoids, except for isoflavones, have an aromatic ring attached at C2 of the pyrone, whereas in isoflavones it is attached at C3. Flavones and flavonols have a double bond at C2 and C3, but flavonols are hydroxylated at C3 position, and once they are oxidized, they become their respective dihydroxylated forms; flavanones and flavanonols. A more extensive review on the structural characteristics of flavonoids can be found in this issue [16].

The levels of flavonoids can significantly vary between different plants [17] and tissues and can reach ~70% of the dry weight in some fruits [18]. Flavonoids have critical roles in plant regulation in response to biotic and abiotic stress. Flavonoids provide protection against pathogens [19,20,21,22], UV light [23], regulate plant fertility by modulating cellular transport of auxin [24], stimulate pollen germination [25,26], and mycorrhizal symbioses by promoting the colonization of nitrogen-fixing bacteria and mycorrhizal fungi [27]. Some plants produce flavonoids as an acclimation process to environmental stressors [28,29,30,31]. Thus, foods obtained from different climatic conditions may differ on flavonoid content based on temperature, day length, and solar radiation exposure [32]. Flavonoid biosynthesis is regulated in plants by the interaction of the R2R3-type MYB domain family of transcription factors, basic helix-loop-helix, and regulatory proteins (WD repeat protein) [33]. The WD repeat protein is constitutively expressed while the bHLH protein is critical for regulating flavonoid biosynthesis [34]. Stress conditions such as ultraviolet-B light have been shown to induce the production of quercetin and luteolin over kaempferol and apigenin in *Ligustrum vulgare* (wild privet) leaves [35,36]. 

Classically, flavonoids have been considered effective antioxidants. The dihydroxy B-ring-substituted flavonoids have been found more effective at quenching reactive oxygen than monohydroxy B-ring compounds [37]. Emerging roles of flavonoids as direct modulators of lipids, nucleic acids and proteins might unravel new functions in modulating gene expression and RNA metabolism [38,39,40]. Flavone, or flavonol glycosides, were regarded as very effective antioxidants [41]. In plants, flavonoids, with the exception of flavanols, are usually found conjugated with various complexity of monomers or dimers of sugars such as rhamnose, glucose, galactose, xylose, apiose, or arabinose at positions 3 and 7 on the A ring for some flavones, flavanones, flavonols, anthocyanins, and isoflavones, while flavone are also conjugated at the 5, 6, and 8 positions, as shown in Figure 1 [42]. Glycosylation of flavonoids improves their photostability and water solubility of the flavonoid aglycone forms; hence, it facilitates their retention in vacuoles [27,43]. Flavonoids are often found as *O*-linked glycosides or as the *C*-glycosides in storage vacuole of grains such as maize, wheat, buckwheat, and rice [44]. Importantly, studies from our group showed that glycosylated flavonoids have less immune-regulatory activity in macrophages and in vivo when compared with their aglycone counterparts, due to their limited cellular absorption, suggesting the relevance of the removal of sugars as a strategy to increase bioavailability [45].

The complexity and variety of glycoside combinations contributes to the vast chemical diversity of flavonoids, with the exception of flavanols, which are not covered in this review (Figure 1). Some glycoside arrangements are more common than others within a given food ingredient, becoming the chemical signature of that particular flavonoid family. For instance, quercetin-3-*O*-rutinoside is the most abundant flavonol in tea, while quercetin-4′-*O*-glucoside, quercetin-3-*O*-rutinoside, quercetin-3-*O*-diglucoside, and kaempferol 3-*O*-glucosides are the most prevalent forms of flavonols in vegetables [46,47]. Capers have the highest concentration of flavonols (234 mg/100g) than any other food and are abundant in kaempferol 3-rhamnosyl-rutinoside, quercetin 3-rutinoside, and kaempferol 3-rutinoside [48]. Similarly, flavones are abundant in chamomile, parsley, celery, and citrus peels. Specifically, chamomile is abundant in apigenin-7-*O*-glucoside and carboxylated with malonyl, acetyl, and caffeoyl derivatives [49] while apiosylglucoside malonyl is found in parsley [50]. Luteolin 8-*C*-glucoside was found in various teas such as rooibos and green tea as well as luteolin 6-*C*-glucoside in green and black teas [51]. Citrus juices were found to have both flavone *O*- and *C*-glycosides such apigenin-7-*O*-glucoside and diosmetin 6,8-di-*C*-glucosides [52] as well as flavanones such as hesperetin-7-*O*-rutinoside and naringenin-7-*O*-rutinoside [53]. Less common food flavonoids, such as isoflavones and anthocyanins, are found abundantly in soybeans and berries, respectively. Selecting foods rich in the flavonoid of interest is equally as important as using foods with a specific flavonoid chemical profile.

Flavonoids have long been recognized as exerting health beneficial effects. The health benefits of flavonoids have been attributed to a number of biological activities, including anti-tumor, anti-metastatic and anti-inflammatory. Flavonoids also act as signaling molecules modulating cell growth, inducing apoptosis, and reducing reactive oxygen species production, presenting potential alternative approaches for treatment and prevention of cancer [54]. The position of the double bond in flavanones was found to inhibit the activity of the breast cancer resistance protein (BCRP) [55]. Moreover, methylation of A and B rings are found in many of the different flavonoids in plants [56,57]. Methylated flavones acacetin, diosmetin, and chrysoeriol glycosides as well as methylated flavanone and hesperidin is found in citrus fruits [52,58,59]. Methylated forms of isoflavones, formononetin, biochanin A, pratensein, and prunetin, are largely found in red clover [60,61]. The methylated isoflavone, biochanin A inhibits aromatase activity when a significant reduction in CYP19 mRNA abundance was found in estrogen receptor-negative breast cancer cells [62]. Structure and function studies report methoxy group substitutions at C7 and C5 position in flavanone and C5 in flavones showed anti-proliferative activity in MCF-7 tumor breast cancer cells [63,64]. Evaluating the effect of the structure in the bioavailability and efficacy of flavonoids in human clinical studies will warrant a better understanding of the potential use of flavonoids in cancer prevention and treatment. 

## 3. Agricultural Engineering to Enhance Flavonoid Content in Food Material

In recent decades, in order to improve the value of food crops towards human health, there has been a shift from selecting crops on their agronomic merits towards selecting crop breeds to enhance the quality and quantity of flavonoids. Cultivar selection and strategic modification of biotic (rhizosphere and soil microbiome) and abiotic (UV exposure, and temperature) stress can be used to enhance flavonoid content in food crops [60,65,66,67,68,69,70]. Differences in flavonoid levels were reported to be up to five-fold in specific strawberry cultivars and almost a doubling of flavonoid content has been found in compost-treated soils compared with fertilized soil [71]. Similarly, soy isoflavone content has been shown to vary as much as 30% between cultivars and as much as 50% depending on soil conditions and location [72]. Exposure to ultraviolet-B light and warm growing temperatures have shown to stimulate the accumulation of flavonoids [73]. Flavonol content of tomatoes grown in Spain, Israel, and South Africa is five times higher than those grown in greenhouses in the United Kingdom [74]. Blue light transmitted by light-emitting diode (LED) was shown to increase the quantity of phenolic compounds in fruits and vegetables. Studies using colored nets or LED lights to transmit blue light increases the production of anti-oxidants and scavenging activity [75]. LED was used to transmit blue light on red lettuce, increases in quercetin-glycosides was observed [76]. Flavonol and anthocyanin content was measured in raspberries at various stages of maturation. Both kaempferols and anthocyanins increased with increasing maturation, while quercetin diminished during maturation [77]. Overall, the selection of varieties of crops and vegetables as well as the optimal growth and processing conditions are key to strategically increase flavonoid accessibility.

## 4. Dietary Intake of Flavonoids and Their Epidemiological Relevance in Food Design

The global variation in the quantity and the types of flavonoids consumed is influenced by differences in lifestyle, cultural food habits, gender, and socioeconomic status which collectively with environmental factors contribute to differences in disease prevalence [78,79,80,81]. Worldwide, the intake of dietary flavonoids is between 50 and 400 mg/day [82,83,84,85,86,87,88,89,90,91,92,93,94,95,96,97,98,99,100], Table 1. In recent decades dietary intake of flavonoids has evolved from limited number of flavonoid groups such as flavones and flavonols [84,87] to include flavonones, anthocyanin and isoflavones, as well as their polymeric forms (procyanidins and thearubigins) [82,83,85]. 

The predominant sources of total flavonoids in Westernized countries, specifically Australia, US, and Europe are tea, citrus fruit or vegetable juices, and wine, with estimated intake ranging from 200 mg/day in Australia [83] to 500 mg/day in Europe where tea consumption is high [88]. In countries such as Spain, Poland, Mexico, and Greece where diets are rich in citrus and wine, dietary flavanones can range from 30 mg/day (Greece) to 170 mg/day (Poland) [88,97,98,99]. Whereas dietary flavanone intake in other Westernized countries can be as low as 6 mg/day in Australia, ~15 mg/day in the US, and ~40 mg/day in Europe [83,84,85,89,90]. Similarly, Asian countries (China, Japan, and Korea) dietary flavanone intake ranged from 36 mg/day (Korea) to 5 mg/day (China) [86,91,92,93]. The total daily intake of flavonoids among the three Asian countries ranges from 90 to 196 mg/day. Flavonol intake was reported to be similar worldwide ranging from 15 to 50 mg/day [82,83,84,85,86,87,88,89,90,91,92,93,94,95,96,97,98,99,100].

The daily isoflavone intake was strikingly different between Westernized and Asian countries. Dietary isoflavone intake in Europe and the Americas is ~1–2 mg/day compared to Asia, where it ranges from ~10 to 60 mg/day [82,86,93,94,99,100]. High isoflavones intake has been associated with a 60% reduction of breast cancer risk in Chinese women, particularly those with high waist-hip ratios and body mass indexes greater than 25 kg/m^2^ [101]. Isoflavone intake in recent meta-analyses and case-control studies have shown to have a protective effect against lung, breast, ovarian, and bladder cancers [8,102,103]. However, no association between breast cancer and total intake of isoflavones was found in studies of Europeans cohorts [104]. These divergent results might be attributed to genetic variation, relatively low intake of isoflavones in Europeans or differences in processing of the soybean foods in the different regions. Asian diets favored consumption of less processed and whole soybeans as compared as Westernized countries [105,106]. Flavone intake varies also significantly worldwide, ranging between 1 to 10 mg/day in Asia and Europe, respectively [85,86]. In Southern Europe, where the Mediterranean diet behavior is frequently practiced, the flavone intake can reach 10 mg/day. This increased intake of flavone in Italian women was associated with a decrease risk in breast cancer, specifically in pre/peri-menopausal women [107]. An Italian case-control study reported that flavones show a protective effect on bladder cancer [103]. Moreover, a meta-analysis combining 12 independent studies reported a significant association of flavonol and flavone intake with a reduced risk of breast cancer was found specially for post-menopausal women, however no significant associations was found with intake of flavan-3-ols, flavanones, anthocyanins or total flavonoids [108]. Similarly, a meta-analysis conducted by Grosso and colleagues identified a possible decrease risk in breast cancer associated with total flavonoids, flavonols, and isoflavones intake; yet, the risk of breast cancer increased with dietary flavanone intake [8]. Other epidemiological studies report that hesperidin was positively associated with lung cancer in smokers [109,110,111]. These results highlight the need for additional epidemiological studies involving dietary flavonoid intake in reducing cancer risk as well as the importance of dose-related responses of flavonoids for health benefits to manifest.

## 5. Flavonoid Bioavailability and Metabolism: Fundamental to Their Bioactivity and Efficacy

The bioavailability of flavonoids is determined by the levels on which they are found in the diet, solubility, absorption metabolism, and excretion. In cellular models, effective concentrations of flavonoids are usually high ranging from 50 to 200 μM [112,113]. These levels are nearly impossible to achieve using conventional food products in dietary interventions. Studies using dietary flavonoids in mouse models of breast cancer also offered promising effects, yet most of them use pure aglycones consumed as part of the diet. However, the use of aglycones in human clinical dietary intervention studies is not feasible because of their poor solubility (aglycones more readily absorbed but glycoside forms are more soluble), expense, and chemical difference from those in the diet. Moreover, to improve the effect size and minimize heterogeneity in animal studies, single gender (typically males), inbred rodent strains with low genetic diversity are often used and placed on well-controlled diets where the flavonoid incorporation can reach supra-dietary levels when a single flavonoid compound or a flavonoid extract is used. The use of whole food ingredient in animal studies presents unique considerations. Food ingredients may have variability due to differences in growth conditions and the food bulk limits their incorporation to be no more than 30% of the diet [114]. Similar challenges occur in human dietary interventions, which may contribute to mixed effects in cancer studies. Some studies with soy isoflavones show an adverse effect on breast cancer (largely postmenopausal, ER positive, HER2 negative, and T_1_ tumor stage number) [115], most show no effect (healthy pre- and postmenopausal at high risk for breast cancer) [116,117], while green tea showed beneficial effects in postmenopausal women with either hormone receptor positive or negative breast cancer [118,119]. The associations between dietary flavonoids intake and breast cancer risk have been reported in several clinical studies. In a case-control study with an American population, a decreased breast cancer risk was associated with an increased consumption of total flavonoids, flavones, flavonols and flavanols, but not flavanones and anthocyanidins [120]. A compatible result of decrease in breast cancer risk with increasing intake of flavones and flavonols were found in case-control studies performed in Italy and Greece [107,121]. Almost similar studies were performed in Mexican population, showing the protective effect of high dietary consumption of flavones and flavonols against breast cancer [122]. An inverse association between the increased consumption of total flavonoids, flavonols and flavanols and breast cancer risk was reported in a French cohort study [123]. However, the results from many other cohort studies in different populations showed fewer clear effects of flavonoids in breast cancer. Thus, there is a great need to conduct additional studies to confirm the beneficial role of flavonoids. 

Mechanistic studies in cell and animal models have shown sound evidence of the immunomodulatory effects of flavonoids in cancer [124,125,126,127]. Flavones show effective immunomodulatory activity at concentrations lower than those required to kill cancer cells [128,129]. The ability of flavonoids to regulate inflammation, a hallmark in cancer, presents new opportunities to control immune cells found in the tumor microenvironment (TME) [130]. The TME is a dynamic ecosystem surrounding the tumor tissues consisting of extracellular matrix (ECM), vascular networks and numerous cell types including stromal cells, fibroblasts, myofibroblasts, adipose cells, and immune cells, among others. A permissive TME environment can enhance tumor cell progression and metastasis [131,132,133,134]. The pathologically increased inflammation in the TME has been shown to be an etiologic factor in many types of cancers. The TME has been shown to have deregulated metabolic properties [135]. Thus, remodeling TME by nutrients may provide a significant option for treatment without any side effects. The metabolic deregulation in the TME exerts an immunosuppressive effect on natural killer (NK) cells [136]. In addition, there are other immunosuppressive cell types such as myeloid-derived suppressor cells (MDSCs), T regulatory cells and tumor-associated macrophages (TAMs) that also contribute to tumor immuno-evasion [137,138]. The accumulation of immune-suppressive cells in the TME has been correlated with poor clinical prognosis in several cancers [139,140,141,142]. Thus, dietary interventions targeted to reduce the number of immunosuppressive cells or reactivate their anti-tumoricidal activity to treat cancer has been gaining great attention.

The dietary flavonoids are able to target the TME, by reprogramming TAMs and inhibiting angiogenesis [143]. Reprograming TAMs by vadimezan (DMXAA), a small flavonoid-like compound, increases TNFα production leading to CD8+ T cell activation [144,145]. In a mouse model of hepatocellular carcinoma (HCC) the flavonoid baicalin has been shown to reprogram TAMs by activating the NF-κB signaling pathway [146]. Luteolin, a common flavone, suppressed the transcription factor STAT6-dependent release of the chemokine CCL2, key regulator of TAMs numbers in the TME and also decreased the migration of Lewis lung carcinoma cells [147]. Grape antioxidants can also target NF-κB by inhibiting its DNA-binding capacity to inhibit cancer cell invasion [148]. Dietary supplementation of grape seed rich in proanthocyanidins was found to decrease the ultraviolet B (UVB 280–320 nm) induced skin tumor development involving reduction in oxidative stress, activation of signaling pathways of mitogen-activated protein kinases and NF-κB and immunosuppression via changes in cytokines [149]. Thus, further studies to unravel how dietary flavonoids inhibit tumor growth and immune evasion are just emerging and will need to be further complemented with human clinical trials.

There is a great paucity of well-controlled human studies involving flavonoid-rich food products that cause changes in tumor size or biomarkers of metastasis. Even more limited is the information in cancer trials on the flavonoid bioavailability or their distribution into tumor tissues. Moreover, emerging evidence shows that the timing of exposure to the flavonoids is critical to their efficacy in cancer progression. Human studies involving flavonoid-rich extracts or whole food products have shown modest beneficial effects particularly in late-stage or recurrent cancers [9,150,151,152]. Yet, a significant reduction of mild to moderate precancerous esophageal has been observed in patients consuming 60 g/day of freeze-dried strawberries, as evidenced by a reduction on the histological grade, proliferation marker Ki-67 expression, as well as decrease of inflammatory markers nitric oxide synthase (iNOS) and phospho-S6 (pS6) kinase [153]. Similarly, black raspberry anthocyanins when administered locally as a black raspberry rectal suppository significantly decreased cellular proliferation, DNA methylation methyl transferase 1 protein expression, and p16 promoter methylation in patients with familial adenomatous rectal polyps after a nine month intervention. These studies show the importance of careful cohort selection and that an insightful study design is a requirement to elucidate definitive benefits of flavonoids in cancer therapy. To date, due to the limited number of dietary intervention trials, heterogeneity in food preparation/compound characterization, and variations in clinical endpoints, a clear consensus regarding treatment duration, dosage, mode of administration, and cohort selection of flavonoids in cancer management has not materialized.

**Figure 2:**
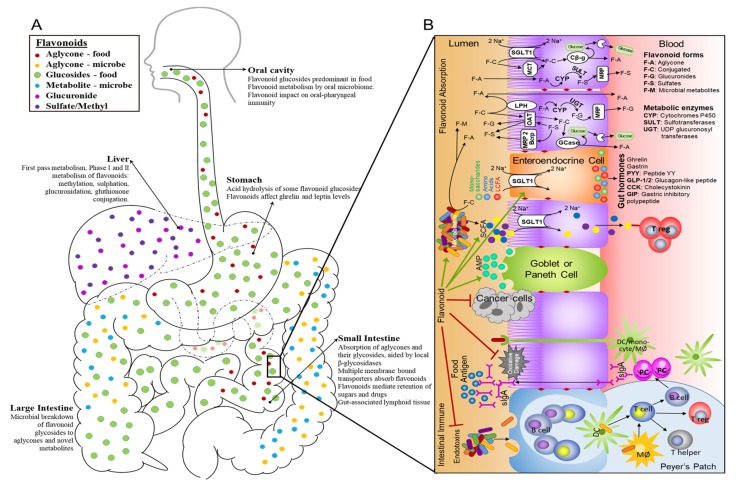
Schematic representation of flavonoid metabolism. (**A**) Flavonoid metabolism and distribution. (**B**) Flavonoid absorption and mechanism of action in intestinal immunity. LCFA: long chain fatty acid; SCFA: short chain fatty acids; DC: dendritic cells; PC: plasma cells; MØ: macrophage.

Flavonoid aglycones are considered to be more bioavailable than their glycosylated forms in vivo since they are absorbed by passive absorption into the intestinal epithelium. In fact, our own studies found higher levels of apigenin in cellular systems and mice fed celery-based diets with deglycosylated flavonoids [45]. Food flavonoids, except for flavanols, exist predominantly conjugated to a variety of sugars and some are better suited than other forms to deglycosylation by β-glucosidase (Figure 1). Studies have shown that flavonoids were prone to metabolism and bioactivation as early as the oral phase of digestion, largely from β-glucosidase activity of the oral microbiome [154,155,156]. Hence, providing beneficial effects on specialized MALT structures of the oral cavity, antigen presenting cells of the oral epithelium, and salivary antibodies in a similar manner to flavonoids on gut immunity [157,158], as shown in Figure 2A. In the recent decade, three intestinal epithelial associated β-glucosidase enzymes have been identified to facilitate the conversion of flavonoid glycosides to their aglycone forms. They include a broad-specificity cytosolic β-glucosidase (Cβ-g) [159], lysosomal glucocerebrosidase (GCase) [159,160], and a brush border bound lactase phloridzine hydrolase (LPH), as shown in Figure 2B [159,161,162]. 

When flavonoids enter the stomach, gastric acids decarboxylate or acid hydrolyze a small portion of glycosides (Figure 2A). Flavonoids have shown to inhibit dipeptidyl peptidase IV activity thereby mediating incretin activity [163]. Incretins are a group of gut hormones that are released postprandially to decrease blood glucose. Glucose-dependent insulinotropic polypeptide (GIP) is one incretin that is secreted from intestinal K-cells in response to glucose and fat ingestion, and its main function is insulin secretion [164]. Another incretin is glucagon-like peptide-1 (GLP-1) which is secreted by intestinal L-cells in the distal ileum and colon. Like GIP, the main physiological effect of GLP-1 is to enhance insulin secretion [165]. GLP-1 inhibits gastric emptying, acid secretion and motility, thereby decreasing appetite [166,167]. GLP-1 and GIP are rapidly inactivated by the enzyme dipeptidyl peptidase-4 (DPP-4) [168]. Heptamethoxyflavone, a flavonoid found in rikkunshito, was critical in stimulating of ghrelin secretion after cisplatin therapy in rats [169] and a small human study (*n* = 10) [170]. This regulation of gut hormones, ghrelin, by flavonoids have implications for ameliorating cancer-cachexia [169]. According to several studies, flavonoid glycosides in the small intestines are absorbed into the enterocyte by sodium-glucose transporter proteins (SGLT), as shown in Figure 2B [171,172,173]. Once sequestered, flavonoid glycoside can be transformed into a flavonoid glucuronide by a process of deglycosylation and glucuronidation. Animal studies suggest that intestinal cell glucuronidation of flavonoids has an important role in flavonoid bioavailability [174] thereby implying that enteric recycling may be an important mediator of flavonoid metabolism [175,176]. Enteric recycling involves reabsorption and re-conjugation of intestinally conjugated flavonoids [176,177]. Studies using intestinal Caco-2 cells show that a predominance of flavonoid glucuronides preferentially mobilize to the basolateral side, where they were excreted into the intestinal lumen and subsequently eliminated as feces or reabsorbed as aglycones. Membrane bound transporters, such as glucose transporters (GLUT1, GLUT2, and GLUT4), have been reported to absorb flavonoid glycosides and in some instances, specifically flavonols and flavanones, were important in regulating blood glucose by inhibiting glucose/fructose uptake in intestine as well as in adipocytes (Figure 2B) [178,179,180,181] 

Moreover, multidrug resistance-related protein (MRP) mediates excretion of flavonoid glucuronides and sulfates from intestinal epithelial cells (Figure 2B) [177,182]. Intestinal MRP are ATP-binding cassette transporter proteins found on the basolateral aspect of crypt cells that prevent the accumulation cytotoxic of xenobiotic compounds by facilitating their cellular excretion [183]. MRP have an important role in the development of drug resistance in cancer cells by decreasing the retention of chemotherapeutic agents. Specifically, the MRP (BCRP) in breast cancer cells causes resistance to topoisomerase I or II inhibitors. Flavonoids such as genistein, naringenin, and apigenin have been found to reverse BCRP-mediated resistance [55,184]. Similarly, quercetin was observed to inhibit P-glycoprotein (permeability glycoprotein) gene expression [185]. Another transmembrane protein, organic anion transporter (OAT), is a solute carrier (SLC) located at the apical portion of the intestinal epithelium and it mediates the uptake of bile acids, hormones and drugs [177,186,187]. Flavonoids such as naringenin and quercetin have been reported to hinder the absorption of medications by inhibiting their uptake by OAT [188]. When fexofenadine was co-administered with naringin, or juices rich in flavonoids (apple and orange juices), their plasma concentrations were less than the controls [187,188]. Another membrane transporter, monocarboxylate transporters (MCT1-14) assists in proton-coupled transport of low molecular weight compounds such as pyruvate [189,190]. In cell and animal studies luteolin was shown to inhibit MCT1 mediated uptake [191,192] suggesting a direct impact of flavonoids in metabolic function. 

Once absorbed in the small intestines or later in the large intestines, absorbed flavonoid aglycones will move along to the liver by portal circulation and undergo extensive first pass metabolism, as shown in Figure 2A. Largely in the liver, but also in the small intestines, flavonoid aglycones undergo oxidative and reductive modifications by NADPH dependent cytochromes P450 enzymes (CYP). Studies with human or mice liver microsomes have demonstrated CYP1 enzymes favor hydroxylation at 3′, and 6 carbons (Figure 1) for biotransformation of apigenin to luteolin/scutellarein, kaempferol to quercetin, and naringenin to eriodictyol, as well as demethylation at 4′ carbon for conversion from hesperetin to eriodictyol [193,194,195]. For isoflavones, the gut microbiome and phase I cytochrome P450 enzymes in human liver microsome were responsible for *O*-demethylation at 4′ carbon; hence formononetin is converted to daidzein and biochanin A to genistein [196,197,198,199] Anthocyanidins, or their monoglucosides of anthocyanins, remained unchanged when incubated with rat liver microsomes in the presence of NADPH, which suggests that anthocyanidins are not affected by phase I enzymes [200]. Wilsher and colleagues reported IC_50_ of CYP1 metabolites (luteolin, scutellarein and 6-OH-luteolin) was significantly lower in breast cancer cell lines (MDA–MB–468 and MCF7) compared to apigenin (parent compound) [195]. Similarly, luteolin (metabolite) was found to be more cytotoxic than its parent compound, diosmetin, in hepatic cancer cells (HepG2) [201]. This bioactivation by CYP1 enzymes was also observed with quercetin where the IC_50_ of quercetin (metabolite) in MDA–MB–468 human breast cancer cells were significantly lower compared to kaempferol (its parent compound) [195]. 

The hydroxyl groups of flavonoid aglycones or flavonoid metabolites of phase I metabolism are targets for Phase II enzymes either in the liver or small intestines. Catechol-*O*-methyl transferases (COMT) are responsible for *O*-methylation, phenol sulfotransferase (SULT) for sulphation, and uridine 5′-diphosphate glucuronosyltransferase (UGT) for glucuronidation of flavonoid aglycones [202,203,204,205,206,207]. These conjugates can be mobilized to systemic circulation where they will eventually be excreted in urine or back to the intestine with biliary secretion [177,208]. Therefore, Phase II enzymes play an important role in the distribution and bioavailability of flavonoids as they are involved in various manner of flavonoid recycling (enteric, enterohepatic, and cell-specific) [175,209,210]. The phase II enzymes and their flavonoid metabolic products can mediate their bioactivity in cancer cell lines. Glucuronide, sulfate, and methyl conjugates of quercetin, yet not its aglycone, when incubated with human A549 lung cancer cell line, upregulated PPAR-γ and arrested cell cycle at G2/M phase [211]. Significantly higher levels of luteolin aglycone were observed in rats administered with luteolin in the presence of LPS than on those receiving luteolin alone, suggesting that conjugated flavonoid metabolites residing as stable precursors in circulation are activated by degluronidation from ionomycin/cytochalasin B stimulated neutrophils during inflammation [212]. Localized gluronidase activity has been observed in neutrophils, macrophages, and colon [210,212,213]. 

Both the diversity of gut microbiome and complexity of flavonoid glycosides and their metabolites further complicate flavonoid metabolism and absorption in the large intestines. The gut microbiome can deglycosylate both simple and complex flavonoid glycosides into their respective aglycones and the aglycones themselves can be transformed by bacterial disruption of the *C*-ring, leading to the production of novel phenolic metabolites. [214,215,216,217,218]. Frequent phenolic metabolites of flavanol, flavone, flavanone, and anthocyanin are methyl and/or hydroxyl derivatives of benzoic, coumaric, ferulic, gallic, hippuric, phenylacetic, (phenyl)propionic, syringic, and vanillic acids [53,200,214,219,220,221]. Phloroglucinol, protocatechuic acid (PCA) and phloroglucinaldehyde (PGA) have also been frequently reported as metabolites of flavonones anthocyanins [219,222,223,224]. Whereas equol, ODMA (*O*-desmethylangolensin), 6-OH-ODMA are metabolites unique to isoflavones [215,225,226]. 

The interplay between microbiome and flavonoid metabolism in the colon as well as our understanding of gut immunity and the brain-gut axis are emerging to have an important role in health [223,227,228,229]. Flavonoids such as apigenin, anthocyanins, and isoflavone metabolites (equol and *O*-desmethylanglolensin—ODMA) have shown to suppress appetite hormones, pro-opiomelanocortin/cocaine and amphetamine-related transcript, glucagon-like peptide 1, and leptin, respectively [230,231,232,233,234]. Likewise, cohort studies in the US population identified an inverse association in total flavonoid intake with body mass index, waist circumference, and inflammatory markers [235,236], specifically with anthocyanins and flavonols [237]. Low molecular weight phenolic compounds (once regarded as degradation products) as well as novel flavonoid metabolites of colonic microbiota have shown to have bioactivity equal or greater than their parent compound [238,239,240]. Among the many flavonoid metabolites, equol, a mammalian metabolite of daidzein, showed bioactivity much greater than its parent compound. Permanent sterility in ewes from uterine hypertrophy caused by dietary red clover isoflavones lead to the discovery of equol in sheep. [241,242]. Equol has shown to have protective effects on breast cancer [243,244]. Interestingly, the flavonoids themselves have shown to mediate the composition and diversity of gut microbiome through inhibition of pathogenic bacteria and promoting commensal bacterial growth [245,246,247,248,249]. However, most of these studies lack information about the levels of bioavailable flavonoids in plasma or tumor tissues. This gap in the field impinges in the development of effective dietary intervention trials.

## 6. Foods Designed to Enhance Delivery of Bioactive Flavonoid

Delivery of physiologically relevant amounts of bioactive compounds to desire organs is critical when formulating functional foods for targeting disease conditions. Integration of biologically active ingredients into a food vehicle requires an understanding of their release from the food material, deposition to intestinal epithelium (bioaccessibility), chemical stability, and subsequent delivery to target tissues. There are several challenges inherent to flavonoids when designing foods for clinical studies. They have relatively low abundance in food materials, range of bioavailability, susceptible to processing loss, and biological transformation. Collectively, these factors require enormous amounts of specific food ingredients to be consumed for health benefits to appear [173,250,251]. Therefore, food design needs to be structured to reduce the food bulk needed to reach bioefficacy and for clinical feasibility [252]. 

The bioaccessibility of flavonoids from food materials has a critical role in flavonoids bioavailability. Bioaccessibility refers to the proportion of flavonoids that is released from the food matrix into the gastrointestinal tract, which can then become available for absorption by the intestinal epithelium [253,254]. Included in the definition of bioaccessibility is the transformation where the food material undergoes by food processing (homogenization, cooking, and fermentation) [255,256] and digestive processes (mastication and digestive enzymes) [257,258] so that it is ready for assimilation into intestinal epithelium cells and hepatic metabolism via portal circulation. In vitro digestion procedures are often used to assess bioaccessibility of food components in complex food materials [257,258]. Whereas bioavailability is the proportion of ingested nutrient or compound which reaches the systemic circulation and is utilized as manifested by its bioactivity [259] or quantity of compound or their metabolite found in systemic circulation [260,261]. Bioaccessibility of dietary flavonoids are an important consideration in food design since flavonoids are found in diverse plant organs, where they are entrapped under different conditions [262]. Plant cell wall polysaccharides, such as cellulose and pectin, are recalcitrant to digestion in the small intestines and hence entraps flavonoids [262,263]. Therefore, size reduction schemes (i.e., milling, homogenization, compression) in combination with heat, shear, and chemical treatments are needed to release flavonoids from plant cells, as well as juicing, which can be used to separate flavonoids from their dietary fiber to improve flavonoids bioaccessibility [255,256,264,265]. 

Flavonoid glycosides are hydrophilic compounds and their bioavailability are conditioned by the absorption of their aglycones or simple β-glucoside. Early studies investigated the behavior of various flavonoid glycosides to acid and enzyme hydrolysis. Findings from the study suggest that the location of the sugar substitution (C3 being most prone to hydrolysis and C7 was the least), the sugar type (rhamnose most rapidly hydrolyzed while glucuronic acid was the slowest), and the flavonoid structure itself determined the rate of hydrolysis [56]. Among the flavonoids, flavonol-*O*-glycosides can be readily deglycosylated with acidification, juicing, and heat [43] yet with anthocyanidins, acid and acylation of the glucoside stabilize their flavylium cation [266,267,268], as shown in Figure 1. Moreover, *C*-glycosylations are the most resilient to cleavage by enzymes or food processing; hence these types of flavonoid glycosides are presumed less bioavailable than their *O*-glycoside forms [264,269,270]. The bioavailability of *C*-glycosides of apigenin (aspalathin) and luteolin (nothofagin) and four eriodictyol-*C*-glycoside isomers were investigated using unfermented and fermented leaves of rooibos tea [271]. The aspalathin and nothofagin content was 10 times higher, while eriodictyol-*C*-glycoside was four times lower in the unfermented tea compared to the fermented tea, suggesting that *C*-glycoside dihydrochalcone are susceptible to degradation by oxidation (tea fermentation) compared to flavanones. These compounds were not found in plasma. However, following unfermented tea intervention, *O*-methyl-aspalathin-*O*-glucuronide (229 nmol) was excreted in the urine and after fermented tea intervention, eriodictyol-*O*-sulfate (68 nmol) was found in the urine [271]. These observations underscore the importance of utilizing food processing to selectively enhance flavonoid bioavailability. 

In contrast to tea fermentation, which is largely an oxidative process, conversion of glycosides to their aglycones can also be enhanced using bacterial and yeast fermentation. Bacterial fermentation of soy foods (miso and soy milk) have reported 90% of total isoflavones as aglycones [272,273] yet sourdough soy bread had 30% of total isoflavones converted into aglycones [274] and a yeast-fermented soy bread with almond meal achieved 75% of total isoflavones as aglycones [275]. These yeast-fermented soy breads (glycoside-rich soy bread (SB) and aglycone-rich, soy-almond bread (SAB) were evaluated in men with recurrent prostate cancer in a randomized crossover trial over a 56-day period. Our findings showed significantly faster peak serum concentration (T_max_) in plasma daidzein, dihydrodaidzein, genistein, and 6-*O*-hydroxy-*O*-desmethylangolesin (6-OH-ODMA) as well peak absorption (C_max_) of genistein and ODMA was significantly higher after the SAB meal compared with SB [276]. In addition, we observed significant reduction in various plasma cytokines and chemokines [277]. Highlighting the ability of flavonoids to overcome tumor immune-evasion, we observed a significant decrease of monocytic myeloid MDSC (recognize by CD33^+^ HLA DR^-^ CD14^+^ surface markers), accompanied by a significant increase of NK CD56^+^ cells during soy bread consumption [277]. 

Likewise, when whole food ingredients are used, flavonoid glycoside stability cannot be considered static nor in isolation because of the abundance of polysaccharide modifying enzymes, which can be activated and inactivated during food processing [262,278]. Endogenous plant enzymes can facilitate as well as hinder bioaccessibility and bioavailability of flavonoids. Pectinase enzymes were utilized to enhance anthocyanins and total flavonoid content in black carrot juice [279]. However, in orange juice, pectinase inactivation was necessary to stabilize citrus juice cloud [264]. Flavanones and flavones in commercially processed citrus juices were found largely concentrated in the cloud during processing and storage [264], but active pectinases have been shown to destabilize the cloud by causing pectin to precipitate, polymerize with flavonoids, and diminish their bioaccessibility [280,281]. Analogously, these endogenous enzymes can be utilized to catalyze the conversion of flavonoid glycosides to their aglycones. Transformation of malonyl apiin to apiin in parsley or celery juice from fresh was significantly greater compared to juice from steamed celery or parsley leaves, a difference attributed to the presence of malonyl esterase in the fresh vegetable [50]. Further heating and acidification of the celery or parsley juice enhanced the conversion of malonylapiin (apigenin 7-*O*-apiosylglucosides) to 83% apiin (apigenin 7-*O*-glucoside) [50] (Figure 1). However, in soy glycosides, chemical conversion of malonyl glycoside to acetyl glycoside was observed after roasting and conversion to the simple β-glycoside was observed after steaming [275], as seen in Figure 1. In this same study, 74% of the total isoflavones was converted into aglycones when β-glycosidase-rich almond meal was used in combination with bread dough fermentation. Quenching polyphenol oxidase activity with blanching improved anthocyanin bioavailability in blueberry puree [282]. Moreover, total anthocyanin in plasma at 1.5 h after consumption from healthy men was significantly higher with blanched blueberry puree intervention compared to non-blanched blueberry puree [282]. Similarly, in two separate studies, plasma naringenin levels were greater after cooked tomato [283] or tomato sauce [284] intervention compared to raw interventions. In line with these findings, novel naringenin chalcones are found in tomato skin and readily convert into the aglycone form with heat (Figure 1).

The physicochemical interactions of flavonoids within their food microenvironment [256], food matrix [285], and components of the background diet [286,287,288] also have an integral impact on their bioaccessibility and bioavailability. At the microstructure level, flavonoid bioavailability can be hindered when flavonoids aggregate to cell wall polysaccharides [256], bind to cell wall fibers and starches [265,289], and plant proteins [290]. Although food processing methods can be used to promote the release of flavonoid from their food microstructure, they can be tailored to formulate specific food matrices to mediate the rate and site of flavonoid delivery. The food matrix dictates the residence time in the mouth during mastication, regulates gastric emptying, and intestinal transient time, thereby modulating the time of flavonoid exposure to the absorptive surfaces of the oral mucosa, intestinal epithelia, and colon. A combinatorial effect of gender and food matrix was reported for isoflavone metabolite dihydrodaidzein (DHD) and *O*-desmethylanglolensin (ODMA) after soy beverage and soy bread consumption [291]. Similar amounts of total isoflavones (~90 mg/day) were consumed either as soy beverage or soy bread in men and women. DHD and ODMA excretion in urine from women after soy bread intervention was three times greater than men [291]. 

Food matrix can be exploited to design foods to target flavonoid delivery to local tissues or systemically. One strategy is to incorporate flavonoids with food polymers (carbohydrates and proteins) which contribute physical characteristics to foods. These food polymers can be processed to many amorphous states such as a solid (glassy and hard), elastic, or viscous forms and are known to readily release compounds such as flavonoids [292,293]. Since these amorphous forms have very different physical characteristics, the contact time will vary between the absorptive membrane and food vehicle/flavonoid and provide unique absorption patterns at the cellular level [251,294]. For instance, solid mucoadhesive gel was used to localize delivery of black raspberry anthocyanins (cyanidin 3-rutinoside, cyanidin 3-xylosylrutinoside, cyanidin 3-glucoside) to the oral mucosal membrane. This approach was able to deliver all three forms or anthocyanins in the saliva and blood within 5 minutes after their oral application [295]. 

Aside from food processing, various combinations of flavonoids (amongst the various flavonoid groups or within one family) can be used in synergy to enhance bioactivity. Rather than deliver high concentrations of a singular flavonoid compound, food ingredients can be combined to provide a comprehensive profile of flavonoids which extend plasma concentrations of flavonoids, simultaneously affect multiple targets, and promote healthy cell function [296]. For instance, food combinations can be used to direct the activity of membrane transporters to deter excretion of flavonoids. Utilizing various flavonoid combinations provides a broad coverage of flavonoids having different rates of metabolism or production of different metabolites [296]. Currently, the beneficial role of flavonoids or the synergistic effects of the whole food on flavonoid efficacy in cancer remains limited. This is largely due to the lack of well-controlled dietary interventions, which use flavonoid-rich diets long-term and underappreciation in selecting a cohort which would be the most responsive to flavonoid intervention.

## 7. Conclusions

The levels of flavonoids in foods, dose, chemical characteristic, food matrix as well as host factors such as age, gender, nutritional status, and physiologic state have an enormous impact on flavonoid bioavailability. Using a “crops to the clinic” approach together with “targeted food design” will help increase flavonoid content and this could (1) minimize food processing strategies needed to concentrate flavonoid content/enhance flavonoid bioavailability, and (2) improve clinical compliance to the dietary intervention by reducing the bulk quantity and dosing frequency of the study foods. Moreover, food processing can alter the physicochemical properties of flavonoid-rich foods to enhance their bioaccessibility/bioavailability, modulate the rate of flavonoid release to local and/or systemic targets, and promote production of novel microbial metabolites. Innovative use of food processing and novel flavonoid combinations when coupled with well-controlled clinical trials can help advance our understanding of flavonoid bioavailability, inter-individual variation, and the dose disparity among different immune functions. Many environmental and host-related factors affect the immune system sensitivity towards flavonoids and predisposition to disease conditions. The immunomodulatory benefits of flavonoids may not be actualized in healthy individuals, but only when health is compromised, under physiological stress, nutrition deficiency, or disease conditions. An important step to move “flavonoid for health” research into personalized medicine would be to decipher the “window of opportunity” in which “targeted flavonoid food design” will enhance flavonoid-mediated immune responses to help prevent and treat cancer alone or in combination with currently available standard of care.

## Figures and Tables

**Table 1:** Summary of dietary flavonoid intake (mg/day) world-wide.

Country ^a^	Population	Dietary Assessment	Intake (mg/day)						Ref
			Anthocyanin	Flavonol	Flavone	Flavanone	Isoflavone	Total Flavonoid *	
Denmark	*n* = 2822, 41 ± 23 yo	Food record	25	19	3	13		60	[84]
Finland	*n* = 2007, 44 ± 20 yo; *n* = 1575, 41 ± 23 yo	48 HDR ^b^	28 to 126	5 to 17	3	27 ± 43	0.9 ± 3.9	33 to 76	[85,96]
Sweden	*n* = 1210, 41 ± 23 yo	Food record	19	18	2	19		58	[85]
North Europe	*n* = 11,764, 54 ± 20 yo	Food record		24.1 ± 0.4	3.0 ± 0.1	23.6 ± 0.6		51 ± 1	[84]
Belgium	*n* = 1304, 41± 23 yo	24 HDR ^c^	19	19	3	12		53	[85]
Czech Rep	*n* = 1666, 41 ± 23 yo	24 HDR	14	16	4	10		44	[85]
Germany	*n* = 10,419, 41 ± 23 yo	24 HDR	33	27	3	19		82	[85]
Hungary	*n* = 1074, 41 ± 23 yo	Food record	15	23	4	11		53	[85]
Ireland	*n* = 958, 41 ± 23 yo	Food record	9	38	3	8	0.7 ± 1.8	58	[85,94]
Netherlands	*n* = 750, 41 ± 23 yo; *n* = 4085, 48 ± 48 yo	24 HDR; Food record	11	31	2	18	0.9 ± 1.9	62	[85,94]
UK	*n* = 309, 54 ± 20 yo	24 HDR		52.2 ± 2.5	6.7 ± 0.4	51.2 ± 3.7		110 ± 5	[84]
UK	*n* = 1724, 41 ± 23 yo; *n* = 335, 52 ± 12 yo	Food record	16	28	2	9	0.7 ± 1.0	55	[85,94]
Central Europe	*n* = 12,679, 54 ± 20 yo	24 HDR		35.2 ± 0.4	5.1 ± 0.1	40.4 ± 0.6		81 ± 1	[84]
France	*n* = 2278, 41± 23 yo	Food record	28	18	7	10		63	[85]
Greece	*n* = 2687, 54 ± 20 yo; *n* = 200, 60 ± 10 yo	24 HDR; FFQ	13.2	4 to 18	0.8 to 6.4	28 to 43	0.7	52	[84,97]
Italy	*n* = 1513 to 2313, 55 ± 37 yo	FFQ; Food record	17–50	20–56	7–10	20–34	0.1–0.5	67–147	[7,85,94]
Spain	*n* = 410, 41 ± 23 yo; *n* = 7200, 65 ± 15 yo	Food record; FFQ	17 to 39	15 to 80	3 to 40	17 to 130	<0.01	52 to 440	[85,98]
South Europe	*n* = 11,285, 54 ± 20 yo	24 HDR		24.9 ± 0.4	5.6 ± 0.1	33.2 ± 0.6		64 ± 1	[84]
Eastern Europe	*n* = 10,728, 57 ± 12 yo	FFQ ^e^	30 ± 93	106 ± 89	15.5 ± 11	104 ± 70	1.6 ± 0.2	257	[88]
US	*n* = 9801, ≥19 yo	48 HDR	11.5 ± 0.7	15.9 ± 0.4	1.2 ± 0.1	12.2 ± 0.5	0.9 ± 0.1	41.6 ± 0.4	[90]
US	*n* = 8809, ≥19 yo; *n* = 17,900, ≥19 yo	24 HDR	3.1 to 10.0	12.9 to 19.0	1.6 ± 0.2	13.8 to 22.5	1 to 2.6	32 to 55	[82,89]
Mexico	*n* = 115, 315, ≥25 yo ♀	FFQ	18–30	10–14	8–12	32–60	0.5	188–270	[99]
Brazil	*n* = 1103, ≥20 yo	24 HDR; FFQ	6.8 ± 1.1	14.6 ± 0.9	3.6 ± 0.3	16.1 ± 1.9	1.5 ± 0.5	54.6 ± 3.5	[100]
Japan	*n* = 115, 58 ± 10 yo	Food record; WI ^d^		16 to 37	0.3 to 18	10.2 to 48.6	47 to 95	88.9	[91,92]
Korea	*n* = 33,581, ≥19 yo	24 HDR	37.0 ± 1.3	64.6 ± 0.8	1.0 ± 0.1	35.9 ± 2.2	57.5 ± 0.9	196	[86]
China	*n* = 120, 15 ± 3 yo♀; *n* = 2239; 59 ± 3 yo♀	24 HDR, FFQ	9.4 ± 7.9	15 to 25	0.9 to 6.5	0.3 to 25	12.1 ± 11.4	21 to 115	[93,95]
Australia	*n* = 10,851, ≥19 yo	24 HDR	2.9	20.7	0.5	6.7		31	[83]

^a^ North Europe (Denmark, Norway, and Sweden); Central Europe (Belgium, Czech Republic, Germany, Hungary, Ireland, Netherlands, and UK); Eastern Europe (Poland); South Europe (Greece, France, Italy, and Spain); ^b^ 48 HRD: 48 hour diet recall; ^c^ 24 HRD: 24 hour diet recall; ^d^ WI: weighed intake; ^e^ FFQ: food frequency questionnaire; * Total flavonoid does not include flavan-3-ols.
